# A Machine Learning Approach for Wear Monitoring of End Mill by Self-Powering Wireless Sensor Nodes

**DOI:** 10.3390/s21093137

**Published:** 2021-04-30

**Authors:** Vytautas Ostasevicius, Paulius Karpavicius, Agne Paulauskaite-Taraseviciene, Vytautas Jurenas, Arkadiusz Mystkowski, Ramunas Cesnavicius, Laura Kizauskiene

**Affiliations:** 1 Institute of Mechatronics, Kaunas University of Technology, Studentu 56, LT-51424 Kaunas, Lithuania; paulius.karpavicius@ktu.edu (P.K.); vytautas.jurenas@ktu.lt (V.J.); 2 Department of Applied Informatics, Kaunas University of Technology, Studentu 50-214, LT-51368 Kaunas, Lithuania; agne.paulauskaite-taraseviciene@ktu.lt; 3 Department of Automatic Control and Robotics, Bialystok University of Technology, 15-351 Bialystok, Poland; a.mystkowski@pb.edu.pl; 4 Faculty of Mechanical Engineering and Design, Kaunas University of Technology, Studentu 56, LT-51424 Kaunas, Lithuania; ramunas.cesnavicius@ktu.lt; 5 Department of Computer Sciences, Kaunas University of Technology, Studentu 50-210, LT-51368 Kaunas, Lithuania; laura.kizauskiene@ktu.lt

**Keywords:** sensor node, energy harvesting, tool vibrations, tool condition monitoring (TCM), support vector machine (SVM), end milling, piezoelectric transducer

## Abstract

There are many tool condition monitoring solutions that use a variety of sensors. This paper presents a self-powering wireless sensor node for shank-type rotating tools and a method for real-time end mill wear monitoring. The novelty of the developed and patented sensor node is that the longitudinal oscillations, which directly affect the intensity of the energy harvesting, are significantly intensified due to the helical grooves cut onto the conical surface of the tool holder horn. A wireless transmission of electrical impulses from the capacitor is proposed, where the collected electrical energy is charged and discharged when a defined potential is reached. The frequency of the discharge pulses is directly proportional to the wear level of the tool and, at the same time, to the surface roughness of the workpiece. By employing these measures, we investigate the support vector machine (SVM) approach for wear level prediction.

## 1. Introduction

During the machining process, severe tool wear can lead to quality degradation of the workpiece or to the breakage of the tool itself, resulting in unexpected production downtime, or even in damage to the equipment or injuries to the operator [1]. In the production process, more than 75% of equipment failures are attributed to direct tool wear or failure, which accounts for up to 6.8% of the total machining process. Tool change and its wear can also lead up to 3% to 12% of total production costs [2]. The lifespan of the tool depends on a number of parameters, such as lubrication. During milling operation, when lubrication is applied, tool life is estimated to last 75 min, while the process of milling without lubrication cutter tool lifespan is expected to end after 45 min [3].

A generic tool condition monitoring (TCM) system consists of sensors, signal processing, classification and tool condition detection components [4,5]. Sensors are deployed in order to directly or indirectly measure physical signals such as: cutting force, torque, vibration, acoustic emission, current and power, sound and temperature. These physical signals are evaluated for detection of tool wear, chatter or breakage conditions in real-time. General requirements for sensors used in the industrial applications are: low cost, small size, robustness, reliability and non-invasive installation. Sensors that comply with such requirements can be integrated with cloud manufacturing frameworks that enable smart online machining process monitoring, forming cyber-physical systems that are able to learn from data generated by such sensors [6,7]. Such implemented smart tool condition monitoring systems can significantly increase machining productivity and reduce tool costs by optimizing its life. This is achieved by implementing condition-based tool replacement strategies instead of time-based tool replacements, which is especially important in high precision, high speed and complex machining processes.

Implementation of a TCM system in production can ensure early detection of tool wear resulting in a decrease of the production costs as well as increasing production efficiency and ensuring the safety of operators. The use of TCM systems can generate 10–50 % cutting speed increment, reduce up to 75% downtime and 30% maintenance costs. Thus such economic incentives for implementation of tool monitoring systems have led to significant research interest in developing reliable and robust systems to be deployed in industrial environments [4].

To meet the set of requirements for TCM systems to be used in industrial environments, wireless self-powered sensor nodes can be employed. They should consist of transceiver for wireless data transmission, microcontroller for data processing and battery for storing the energy collected from the environment [8,9,10]. Ambient energy harvesting from immediate environment enables increasing the sensor lifespan as well as reducing or eliminating altogether the need for maintenance [11,12]. During milling operation, the common source of ambient energy is the vibration of the tool and/or workpiece, which can be harvested using electromagnetic, piezoelectric, electrostatic and magnetrostrictive principles [13].

The authors of [14] propose the use of an attachable electromagnetic energy harvesting driven wireless vibration-sensing TCM system, which can detect cutter wear and breakage conditions during milling process. Such energy harvester is enough to power a sensor node consisting of power management circuit, three accelerometers and wireless data transmission capability. In [15] the authors discuss the use of a circular bimorph piezoelectric transducer that assures a resonant frequency in the same mode as the turning tool. Such a device is attached to the turning tool collecting vibrations present during operation. Paper [16] discusses the use of bimorph piezoelectric cantilever with an inertial mass attached to a milling tool. The electric energy by the piezoelectric transducer when it is excited by the vibrations of the cutter caused by the impact of its cutting tooth on the workpiece. As the wear of the milling tool increases, the angular acceleration exerted on the tool increases as well, leading to up to two times higher voltage output from the piezoelectric transducer, thus relating the increase in the output voltage to changes in the condition of the tool over time.

The data collected from the sensor node can be analyzed in parallel while the milling operation is in progress by implementing machine learning (ML) algorithms and provide feedback on the condition of the milling tool to the equipment as well as the operator.

The use of machine learning approach for tool wear estimation is being adopted quite quickly in factories where intelligent monitoring systems are being deployed. Since the signals generated by the sensors are non-linear with respect to the tool wear rate, a support vector machine (SVM) model can be used as a classifier to predict the wear of milling tools [17]. Accurate predictions in detecting tool wear under various cutting conditions with rapid response rate are achieved by measuring the audible acoustic signals and analyzing them in the frequency domain by extracting signal features that correlate with the actual milling phenomena. The prediction of the wear of the end mill tool can be considered as the classification task and solved by applying different machine learning algorithms. Tool wear prediction based on linear axis force and current signals using SVM and random forest (RF) approach tend to achieve very good classification accuracy results: 98.1 % (SVM) and 86.1 % (RF) respectively [18]. It can be noted, that employing SVM, tool condition binary classification task into “sharp” and “dull” classes allows to achieve classification rate of 100% [19], using features extracted from three-axis cutting measuring forces, torque, three-axis accelerometer and acoustic emission signals.

Other commonly applied ML methods to tackle this problem include artificial neural networks (ANN), RF, decision trees (DT) [20], neuro-fuzzy systems [21], or convolutional neural network (CNN) [22], with a focus on prediction accuracy and training time. A neuro-fuzzy system (with a feedforward backpropagation neural network) can be used to perform online tool wear condition monitoring by measuring three parameters—maximum tool wear, machining time and cutting power that are required to create a certain surface roughness, thus making the most efficient use of the cutting tool [21]. Deep convolutional neural networks have achieved state-of-the-art results in many imaging recognition tasks, therefore they are increasingly applied to make predictions analyzing tool wear images [22,23,24]. It could be a very efficient method to exclude relative numerical features as well [25,26,27]. However, the provided accuracy results of different ML methods vary considerably, ranging from 50% to 100% [18,19,21,28], depending on the derived features, experimental conditions, the prediction task (classification or regression), the parameters of the ML model and etc. Therefore, it is difficult to provide objective insights or to perform an unbiased comparative study.

The support vector machine—regression (SVR) approach is used to predict tool wear condition. During milling, tool wear changes the surface roughness of the workpiece and therefore the surface roughness values are used as an indirect measure of the tool wear condition. The corresponding empirical and newly derived attributes have been calculated from the original data, i.e., the variation of the capacitor charge level over time, obtained from the proposed sensor node. Common time-series features such as running averages, autocorrelation and entropy were calculated as well. The ten most valuable features were selected for SVR model training. Among the three types of kernels, the prediction model with a radial basis function (RBF) kernel was the best for predicting the value of the workpiece surface roughness. The SVR-RBF model reveals that it is able to provide the lowest average errors of 2.420% based on mean absolute percentage error (MAPE). Furthermore, the results show a significant inverse correlation between the variance of the capacitor charging time (the length of the capacitor charging cycle) and the surface roughness of the workpiece.

The paper is structured as follows: After the introduction, Section 2 presents a method for converting the rotational vibrations of a shank-type rotating tool into longitudinal vibrations by embedding it into a cone-shaped horn with helical slots in the spindle of the machine tool. The advantages of embedding such a tool compared to a conventional one are demonstrated in Section 3. Experimental results on the prediction of tool wear condition using the SVR approach are provided in Section 4. Additional experiments, including time serious features and different machine learning algorithms, are presented in Section 5. Finally, Section 6 concludes the paper.

## 2. Material and Methods

### 2.1. Design of a Horn-Type Waveguide with Helical Slots

A cone-shaped tool holder, also referred as horn, is usually used in machining processes where the excited vibrational response of the tool reduces cutting forces and improves the surface quality of the workpiece. The cone-shape of the tool holder was chosen because it would act as a concentrator-resonator of ultrasonic vibration energy and be as rigid as possible for lateral loads; other shapes (stepped, exponential, catenoidal and etc.) are less rigid, and the helical grooves formed on their lateral surfaces would further reduce their lateral stiffness and be less efficient. The motion generated at the tool-workpiece interface is typically longitudinal, torsional or a composite of longitudinal and torsional (L&T). It can be achieved either by the use of a transducer capable of synchronously generating both vibrational modes simultaneously, or by the introduction of geometric features on the surface of the horn waveguide that enable transformation of longitudinal motion by the transducer into L&T form at the tool.

**Figure 1:**
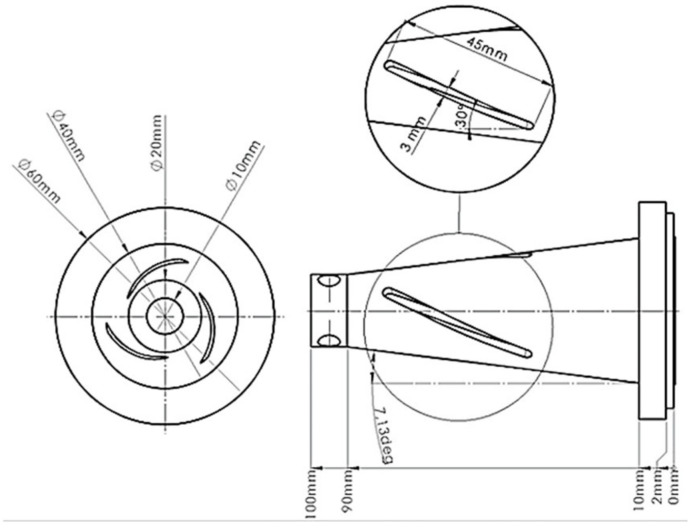
Horn type tool holder model design with helical slots formed on its planar surface.

In this study, a cone-shaped type tool holder design with helical slots formed on its planar surface has been developed using the Solidworks (Dassault Systèmes SolidWorks Corporation, Waltham, MA, USA) computer-aided design (CAD) software package. As presented in Figure 1, the designed cone-shaped tool holder has three uniformly distributed helical slots of 45 mm length, 3 mm width and 3 mm depth, with an angle of 30° on its planar surface with the longitudinal axis of the tool holder. The selected material for the tool holder is C45 steel (EN 1.0503) whose chemical composition and mechanical properties are provided in Table 1.

Introduction of helical slots on the surface of the cone-shaped tool holder enable coupling of L&T vibrational mode in the 14.1 kHz and 15.2 kHz vibrational frequency bandwidth, thus creating an L&T vibrational mode. This L&T vibrational mode can be used in a reverse action during milling operation as compared to the traditional application of horn-type waveguides with helical slots. In our design, when the end mill tool is excited during milling by torsional vibrations entering or exciting the workpiece, these vibrations are transferred to the tool holder where they are partially transformed into longitudinal vibrations due to the generation of the L&T vibration mode. The longitudinal vibrations generated in the tool holder are transferred to an axially poled piezoelectric transducer, where they are used to deform it, thus generating an electric charge.

**Figure 2:**
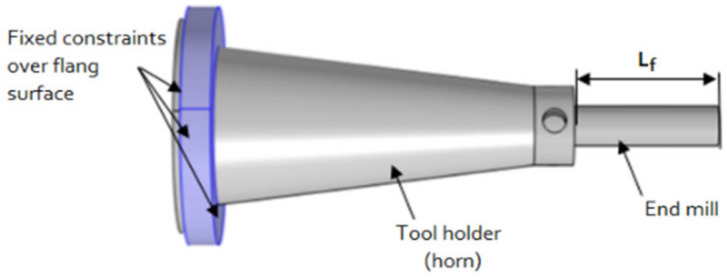
Tool holder with end mill tool geometrical model—rigid fix constraint boundary conditions.

The assembly of a cone-shaped tool holder with an end milling tool made of high speed steel (HSS) material is presented in Figure 2. The tool holder is rigidly attached to the entire outer flange surface. Such configuration was used in subsequent FEM modeling work.

### 2.2. Simulation of Horn-Type Waveguide with Helical Slots

**Figure 3:**
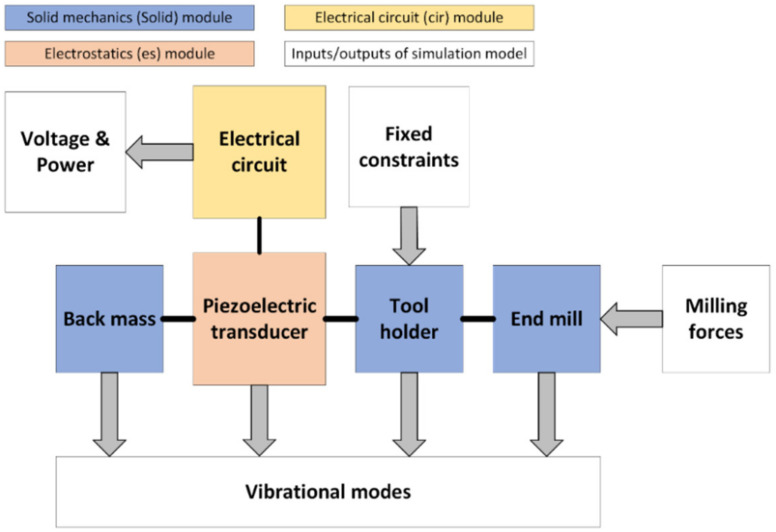
Principal block diagram of the FEM simulation model.

The COMSOL Multiphysics (COMSOL, Inc., Burlington, MA, USA) software package was used to model the vibration response of the horn type tool holder with helical slots. In FEM modeling, the flange surface of the tool holder was firmly attached (Figure 2), and the milling forces were applied to the end mill tool, the principal block diagram of the created FEM simulation model is provided in Figure 3. The evaluation of the vibrational modes and the conditions for the longitudinal-torsional mode coupling effect to take place for the tool holder model and the frequency dependence of the L&T modes on the geometrical and material parameters was performed by modeling. The analysis of the surface displacements of the contact surface of the toolholder with the piezoelectric transducer was carried out in the Solid Mechanics (solid) module in the frequency domain. The complete simulation of the tool holder with the piezoelectric transducer, was performed by integrating the Electrical Circuit (cir), Electrostatics (es) and Solid Mechanics (solid) modules.

In such applications, the motion generated at the tool-workpiece interface is typically longitudinal, rotational, or longitudinal and rotational, which can be achieved either by using a transducer that can simultaneously generate both vibration modes, or by introducing geometric elements on the tool holder surface that allow the transducer to transform the longitudinal motion at the tool into L&T form.

In the considered FEM formulation, the dynamics of the tool is described by the following equation of motion in block form, taking into account that the fundamental law of motion is known and defined by the node displacement vector $u_{K}$:

(1) $$\left[\begin{array}{cc} M_{NN} & M_{NK}\\ M_{KN} & M_{KK} \end{array}\right]\left\{\begin{array}{c} {\ddot{u}}_{N}\\ {\ddot{u}}_{K} \end{array}\right\}+\left[\begin{array}{cc} C_{NN} & C_{NK}\\ C_{KN} & C_{KK} \end{array}\right]\left\{\begin{array}{c} {\dot{u}}_{N}\\ {\dot{u}}_{K} \end{array}\right\}+\left[\begin{array}{cc} K_{NN} & K_{NK}\\ K_{KN} & K_{KK} \end{array}\right]\left\{\begin{array}{c} u_{N}\\ u_{K} \end{array}\right\}\;=\;\left\{\frac{0}{r}\right\}$$

where the node displacement vectors $u_{N}$, $u_{K}$ correspond to the displacements of the free and kinematically excited nodes; M, C, K are the mass, stiffness and damping matrices, respectively; r is a vector representing the reaction forces of the kinematically excited nodes.

The displacement vector of unconstrained nodes is expressed as $u_{N}=u_{Nrel}+u_{NK},$ where $u_{Nrel}$ denotes the component of the relative displacement with respect to the displacement $u_{NK}$, of the moving base. The vectors $u_{N}$ and $u_{NK}$ correspond to the displacements of the rigid body that do not cause internal elastic forces in the structure. The proportional damping method takes the form C=αM +βK, where α and β as Rayleigh damping constants. Thus, by performing algebraic Equation (1), the following matrix equation is obtained:

(2) $$M_{NN}{\ddot{u}}_{Nrel}+C_{NN}{\dot{u}}_{Nrel}+K_{NN}u_{Nrel}=\hat{M}$$

where the left side of the equation contains structure matrices constrained at the nodes of the determined kinematic excitation, and the right side reflects the kinematic excitation applied by the vector of inertial forces acting on each node of the structure.

**Figure 4:**
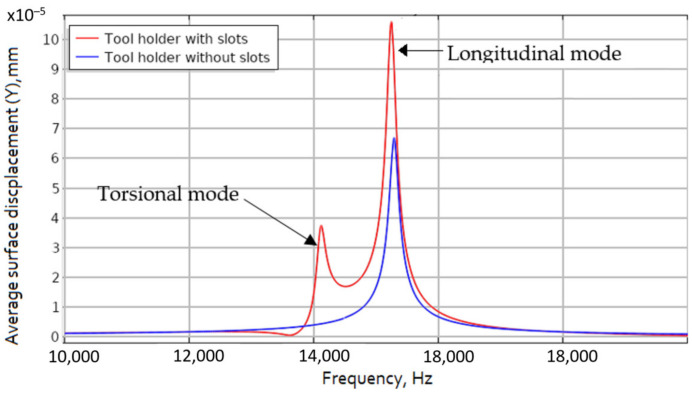
Average displacement amplitude in longitudinal direction for horn with and without slots.

The model verification was performed to validate the adopted tool modeling approach and thus to ensure that the constructed FEM model can accurately predict the dynamic behavior of the vibration-controlled tool. The degree of agreement between the measured and simulated frequency responses was chosen as a quantitative criterion describing the accuracy of the model. One of the main factors determining the vibrational response of a tool is related to its boundary conditions. The main challenge was to achieve a proper frictional locking of the tool in the gripper. In addition, considerable effort was made to ensure that the dynamic analysis applied a kinematic excitation to the FEM model that corresponded to the actual vibration excitation induced by the tool. During the model adjustment phase, the most suitable values of stiffness $k_{z},\;k_{r}$ and $k_{\varphi }$ were captured: the values of these coefficients were adjusted until a sufficiently close match between the simulated and measured eigenfrequencies was reached. This procedure was performed by conducting a sequence of frequency response analyses in the range of 0–20 kHz with different values of stiffness coefficients. For the frequency response analysis, the displacements of the tool holder surface in the longitudinal direction opposite to the position of the end mill were measured and the results are shown in Figure 4.

The obtained results show that a tool holder with uniformly distributed helical slots formed on its conical surface and excited to resonate at its torsional vibrational mode will result in surface displacements more than six times higher than those compared to a conventional design horn type tool holder without surface modifications. Conversely, if the tool holder with helical slots is excited in the longitudinal vibrational mode, the amplitudes of the surface displacement in the longitudinal direction will be more than twice as higher as those obtained from tool holder without helical slots. Across the full generated L&T vibrational mode frequency bandwidth we can see that the surface displacement amplitudes in longitudinal direction at about 15 kHz are at least twice as large as the results obtained with the tool holder without helical slots. This effect is obtained due to the partial conversion of torsional vibrations into longitudinal vibrations in the tool holder.

Tool holder assembly with a piezoelectric transducer allows to evaluate energy harvesting properties under L&T mode excitation. For this purpose an axially poled piezoelectric (material PZT-5H) transducer of OD×ID×H = 40 mm×32 mm×10 mm dimensions has been selected.

**Figure 5:**
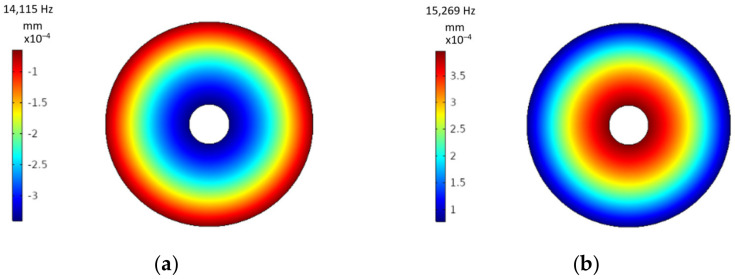
Tool holder with slots surface displacement heat map in longitudinal direction of the transducer at (**a**) torsional, (**b**) axial excitation vibrational modes.

The size and type of this piezoelectric transducer were chosen with respect to the position of the appearance of the maximum amplitudes of displacement of the tool holder flat surface in longitudinal direction, under end milling tool excitation conditions resonating at torsional mode (Figure 5). During milling operation the cutting tool is predominantly excited by torsional forces, thus it is important that the piezoelectric transducer shape would be selected ac-cording to formation of surface displacement at this vibrational mode. According to Figure 5, we can see that maximum surface displacement at torsional mode is formed at the outer diameter of the contact surface, for this reason axially polled, ring shape PZT has been selected to optimize harvesting of vibrational energy.

**Figure 6:**
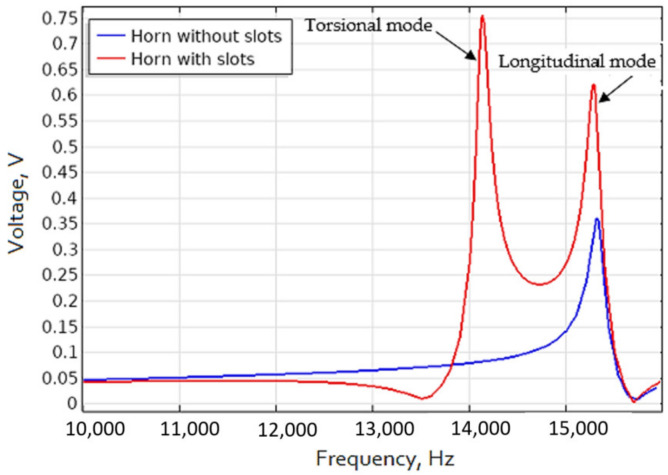
Piezoelectric transducer generated voltage output for tool holder with and without helical slots.

Repeated results of the frequency response of the piezoelectric transducer output voltage in the 20 kHz frequency range are presented in Figure 6. The results show that the transducer, when embedded together with the tool holder with helical slots on its surface, generates a significantly higher output power over frequency range where the L&T mode coupling effect is present, compared to the case where a tool holder without helical grooves is used instead. This confirms the previously obtained results presented in Figure 4, where the highest longitudinal surface displacement is obtained when the tool holder with helical slots is excited to resonate at its torsional mode.

This voltage, generated by the axially poled piezoelectric transducer, can be used to power low-power electronics, such as sensor nodes, which can be embedded inside the tool holder for measuring tool wear parameters during milling operation such as change in capacitor charge over time.

### 2.3. Design of Sensor Node Embedded inside Cone-Shaped Tool Holder for Cutter Wear Monitoring

The voltage obtained from the piezoelectric transducer under deformations when the tool holder is excited to resonance in L&T mode can be harvested by low-power senor nodes. To this end, the design of such a sensor node has been proposed in this study.

**Figure 7:**
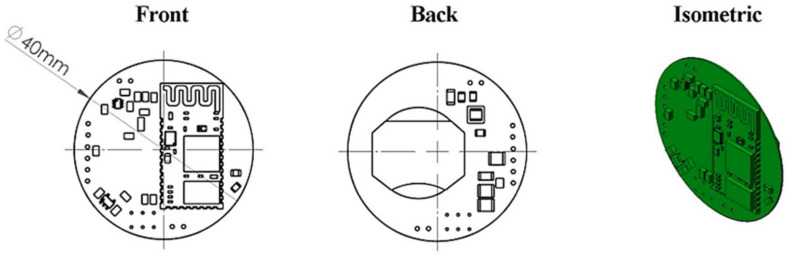
Front, back and isometric views of PCBA with MCU and Bluetooth module. Back view of the designed PCBA shows the introduced placement for coin type battery.

During operation, when the tool holder is excited, the voltage generated by the piezoelectric transducer is fed to electronics assembled as printed circuit board assembly (PCBA). The designed PCBA (Figure 7) consists of power management, data processing and wireless communication units.

**Figure 8:**
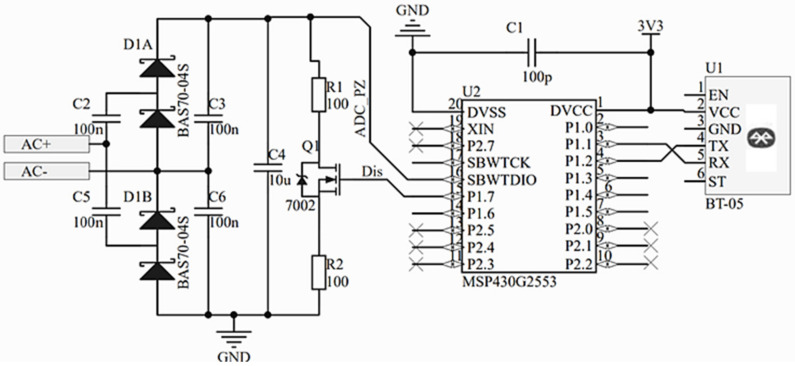
Electrical schematics of the prototype PCBA used with designed sensor.

As the designed device is expected to operate on low power all electronic components have been selected with low power budget requirements in mind. For this reason, an MCU ULP MSP-430G2553 microcontroller (Texas Instruments, Dallas, TX, USA) has been selected. A MLT BT-05 type 4.0 Bluetooth serial communication module is also included for wireless communication with a smartphone. Detailed electrical schematics of the PCBA are provided in Figure 8.

Here, voltage from the piezoelectric transducer generated during milling operation is fed to a voltage multiplier consisting of Schottky diodes (D1A and D1B) and capacitors (C2, C3, C5, C6) where it is converted into a DC signal. From here the voltage is used to charge capacitor C4. The change in the charge level of the capacitor over time is measured by the MCU (microcontroller) and sent via Bluetooth. The denoted charge level change over time is directly related to the vibrations of the tool, which amplitudes depend on the wear state of the tool. As the tool gradually wears out, the amplitude of the torsional vibrations present in the tool during interaction with the workpiece are also increasing [29]. As these vibrations are partially transformed into longitudinal vibrations deforming the piezoelectric transducers, the piezoelectric transducer is subjected to higher stresses during operation with increasing tool wear over time, resulting in higher output voltages. When the MCU measures the capacitor voltage, it also compares it to a set voltage threshold value. In case this threshold value is exceeded, the MCU triggers N-channel field transistor Q1, discharging the capacitor C4 through the resistor loads R1 and R2. Once the capacitor is discharged, another measuring cycle is initiated.

**Figure 9:**
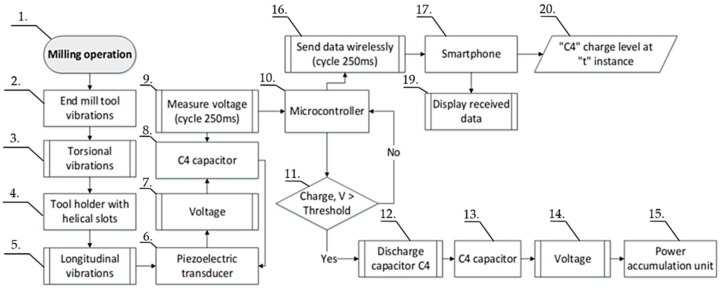
Working process flow of the wireless energy harvesting sensor node used to detect end mill tool condition wear state.

During discharge, the voltage from the C4 capacitor is fed to the power accumulation unit, in our case a super capacitor, where it is stored and used for powering the electronics. This enables the self-powering capability of the sensor node. The principle of operation of the developed device, to be used during milling operation, is presented graphically in the flow chart (Figure 9).

As provided in Figure 9, during the milling operation (1), the predominantly random torsional vibrations exciting the cutting tool (2 & 3) are transmitted to the tool holder with helical slots (4). At the tool holder, these torsional vibrations are partially transformed into longitudinal vibrations (5) and transferred to deform an axially polled piezoelectric transducer (6). The voltage (7) from the piezoelectric transducer (6) is converted into a DC signal and continuously fed to the “C4” capacitor (8). During milling, the charge level of the capacitor “C4” (8) is measured (9) by an embedded microcontroller (10) at every 250 ms time interval. The microcontroller performs the following tasks: it compares the charge level of the capacitor “C4” with a predetermined value (11), in case the measured capacitor charge level exceeds the predetermined threshold, the microcontroller initiates the discharge of the capacitor (12). Here, the capacitor (13) voltage (14) is discharged into the power accumulation unit (15), which is used as a power source by the sensor it-self and the charging cycle of the capacitor “C4” (8) is restarted. In addition to controlling the discharge of the capacitor, the microcontroller (10) also initiates wireless data transmission (16) via Bluetooth to the smartphone (17). The data transmitted contains information on the charge level of the capacitor at the time of measurement. The smartphone is used here to display the received data (19) and to store it on a local hard drive (20) for later processing and analysis.

Thus, the proposed sensor design not only enables the energy harvested by the piezoelectric transducer to be used as an alternative power source of the sensor, but also to measure and record the change of the generated voltage over time, expressed as the change in the charge level of the capacitor. Here, the exponential increase of the capacitor charge level over time can be related to the gradual wear of the end mill tool.

## 3. Experimental Setup

**Figure 10:**
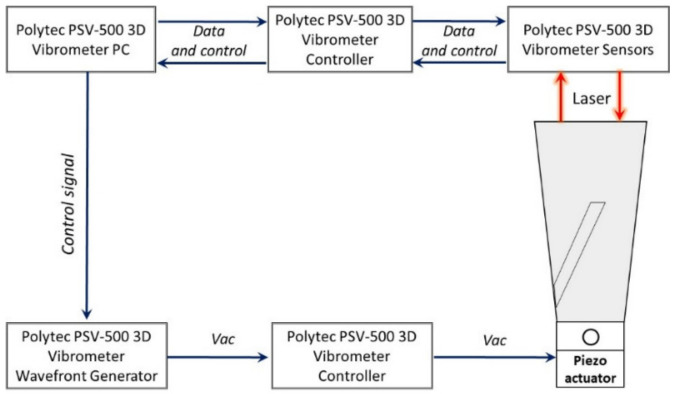
Vibrational response test setup used for tool holder with and without helical slots. (1)—Positioning of the tool holder with and without helical slots during the experiment.

**Figure 11:**
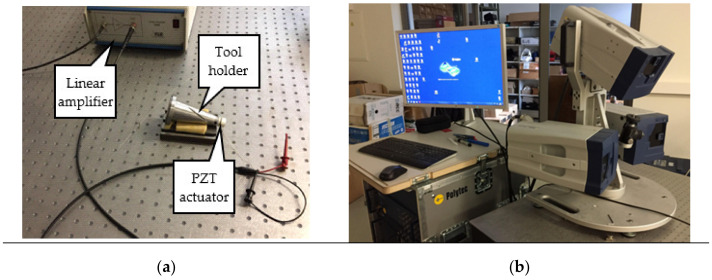
Actual experimental set-up of the vibration response of the tool holder with and without helical slots. (**a**)—view of the tool holder with a fixed piezoelectric actuator and a linear amplifier P200 (FLC Electronics AB, Partille, Sweden), (**b**)—a view of the Polytec PSV-500 3D laser doppler vibrometer-scanner (Polytec GmbH, Widbronn, Germany).

In order to experimentally verify that the use of helical slots on the planar surface of the tool holder lead to higher voltage from piezoelectric transducer, two tool holders have been prepared, one with and one without slots. These two manufactured tool holders was used during vibrational response experimental study as presented in block diagram (Figure 10), while the actual experimental setup is presented in Figure 11. The experimental setup was kept identical for both tool holders.

As presented in the block diagram of the experimental set-up, a piezoelectric actuator was fixed at the end of the tool holder, where the end milling tool is to be mounted to excite the tool holder. The piezoelectric actuator was connected to a waveform generator exciting it by a chirp type signal in the 50 kHz frequency range.

**Figure 12:**
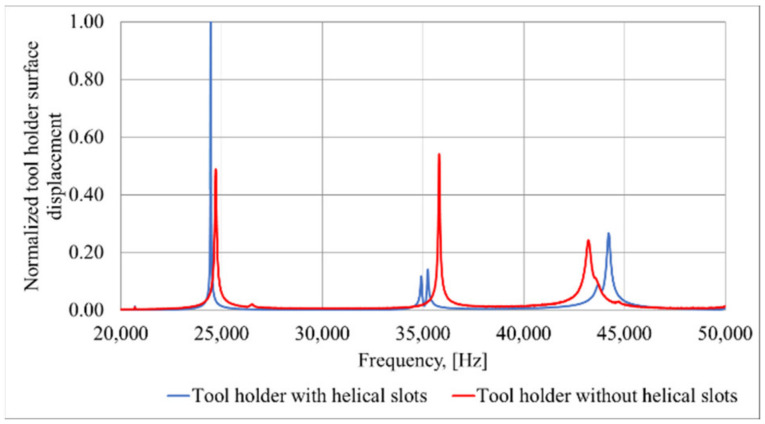
Measured surface displacement amplitudes for the tool holder with and without helical slots excited at axial mode.

A PSV-500 3D laser doppler vibrometer (Polytec, Bake Parkway Irvine, CA, USA) was used for non-contact surface displacement measurements. These measurements were made on the surface of the tool holder, which is dedicated for contact with piezoelectric transducer (opposite position of piezoelectric actuator). Results from the performed vibrational response experiment are presented in Figure 12.

The acquired results show that for the tool holder with helical slots, if it is excited to resonate at its axial mode, the surface displacement amplitude is twice as high, compared to the obtained results for the tool holder without helical slots, when it is excited to resonate at the longitudinal mode. The vibrational response study results are consistent with the results obtained during FEM modeling of the tool holder (Figure 4), showing that under the same excitation condition, the longitudinal surface displacement amplitudes of the tool holder with helical slots are significantly higher when compared to the tool holder without these helical slots. The frequency differences when compared to the FEM model are due to the different mounting position: in the FEM model, the tool holder is mounted to its outer flange surface (Figure 2), whereas in the experimental study it is mounted to its own free weight. Nonetheless, study results show a trend, observed during FEM studies, that the introduction of helical slots on the tool holder lead to the increase of longitudinal vibrations. This is achieved, because the introduction of helical slots enables partial transformation of the torsional vibrations generated at the input surface of the tool holder into longitudinal motion reinforcing the longitudinal vibrations that already exist. These combined longitudinal vibrations are transmitted through the tool holder deforming a piezoelectric transducer.

**Figure 13:**
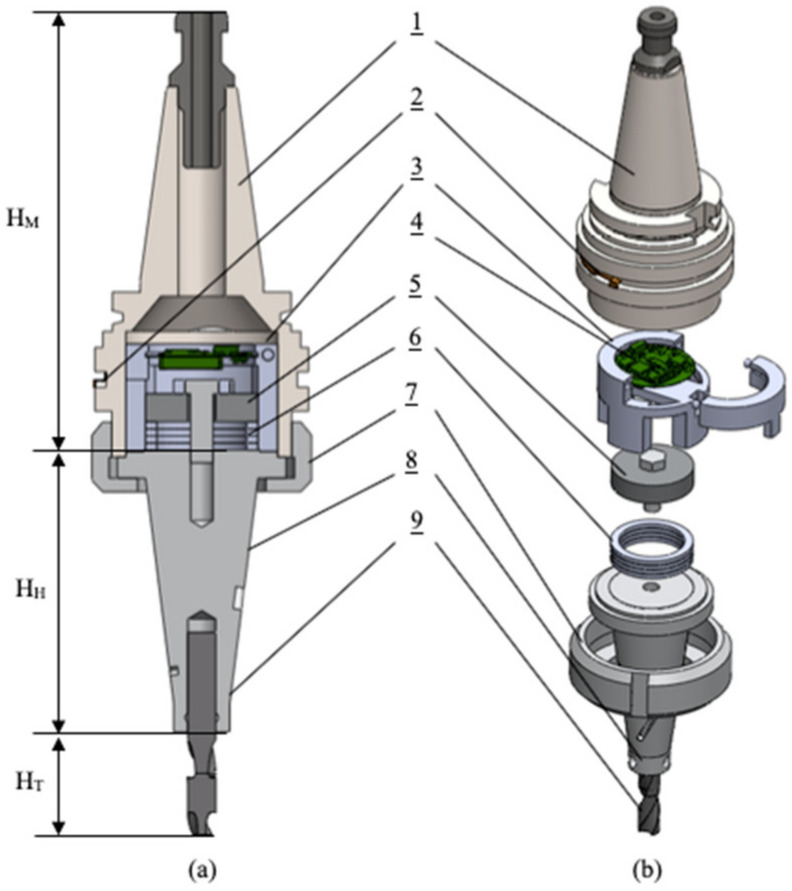
(**a**)—Energy harvester device assembly section view, (**b**)—Energy harvester device assembly exploded view.

For experimental research to monitor the condition of rotating shank-type tools an instrument design was proposed and developed. According to the FEM results obtained in the previous section, the device consisted of a cone-shaped tool holder with three helical grooves uniformly distributed on its planar surface, a piezoelectric transducer and a PCBA board with integrated electronics. A 3D CAD model of the device, designed in the Solidworks (Dassault Systèmes SolidWorks Corporation, Waltham, MA, USA) software, is shown in Figure 13, providing cross-sectional and exploded views and the assembly elements presented in Table 2.

**Figure 14:**
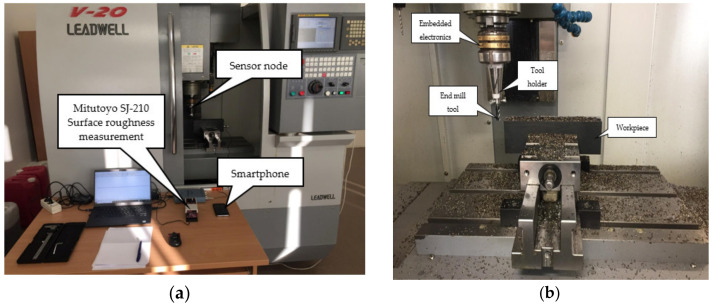
Wireless sensor node used for tool condition monitoring assembled inside Leadwell V-20 CNC milling center (Leadwell CNC Machines MFG., Corp., Taiwan), (**a**)—outside CNC view, (**b**)—inside CNC view.

For the experimental investigation aimed at evaluating the energy harvesting performance of the developed device under actual milling conditions and its dependence on the milling process parameters, the developed device (Figure 13) has been assembled inside the spindle of V-20 CNC milling center (Figure 14, Leadwell, Taichung City 421, Taiwan). This experiment has been repeated for a tool holder with and a tool holder without helical slots on its planar surface. Throughout the milling process, a one-way Bluetooth connection was established with an Android smartphone and the information about the charge level of the C4 capacitor was sent and stored every 250 ms.

The experiments were carried out by machining the entire top surface of a workpiece with the following dimension: length=250 mm,height=50 mm and width=50 mm. The selected workpiece is made from 1.0037 type carbon steel. The chemical composition and mechanical properties of this type of material are provided in Table 3.

The HSS end mill cutting tool was selected for machining the workpiece. The main parameters of the end mill tool are provided in Table 4.

The milling operation parameters were selected according to the workpiece and the cutting tool when used without lubrication, as provided in Table 5**.**

During milling operation, the wireless sensor node was configured to discharge the capacitor C4 if its voltage level reached or exceeded a set threshold of 0.7 V, which would re-set and repeat the capacitor charging process cv.

**Figure 15:**
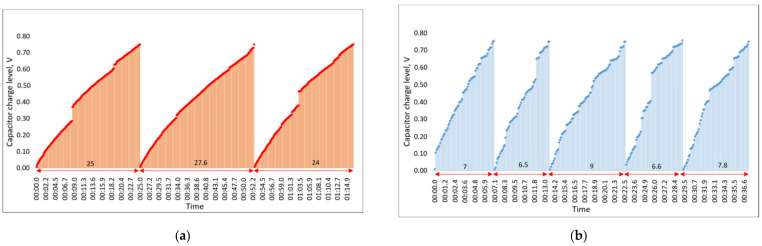
Experimental results of capacitor C4 charging times when tool holder without (**a**) and with (**b**) slots is used during milling operation.

The results of the capacitor charging levels over time, recorded on the smartphone during the milling experiment, are shown in Figure 15.

Figure 15a presents the capacitor charging period during milling operation when the tool holder is implemented without helical slots and Figure 15b shows the capacitor charging rate where the tool holder with three uniformly distributed slots is assembled with our device. From the obtained results, we can see that the average time to charge the capacitor to the set threshold of 0.7 V is 7.38 s when using the tool holder with helical slots and 25.53 s when using the tool holder without slots. The results show that the tool holder with helical slots is charged more than 3.45 times faster, which means that up to 3.45 times more vibrational energy is harvested during milling operation over the same time interval if the device is implemented using the tool holder with helical slots.

**Figure 16:**
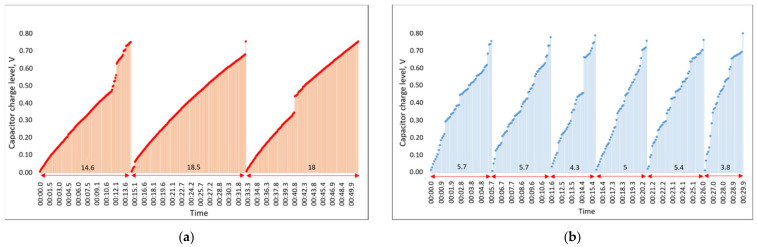
Experimental results of the capacitor C4 charging times when the tool holder without (**a**) and with (**b**) helical slots is used during milling operation, with the depth of cutting increased from 1 mm to 1.5 mm.

In the next step, the experiment was carried out by changing the parameters of the milling process. The spindle speed remained the same at 1210 RPM, but the milling depth was increased from 1 mm to 1.5 mm. The experiment was performed with both tool holders, with and without helical slots, and the results of this study are presented in Figure 16.

The obtained results show that increasing the depth of cut from 1 mm to 1.5 mm resulted in a significant decrease of the capacitor charging time up to the set threshold for both tool holders, with and without helical slots.

The average charging time recorded for the C4 capacitor when assembled with the tool holder without helical slots was 17 s, whereas for the tool holder with helical slots it was 4.9 s. The results show that, as in the last step of the experiment, the difference between the tool holder with helical slots leads to a 3.47 times faster capacitor charging when compared to the one with the tool holder without helical slots.

It is important to note that the charging time of the capacitor C4 decreased significantly with increasing milling depth in tool holders with and without helical grooves, which means that the process parameter has a significant effect on the amplitude of the vibrations excited in the end mill tool during operation. However, the difference in the generated voltage between the tool holder designs remains relatively the same.

The increase in the amount of the harvested energy can be anticipated with the increase in spindle speed, because it leads to the increased frequency of tool tooth contact with the workpiece and hence the frequency of excitation of the milling tool. As the milling depth increases, the cutting edge of the milling tool is subjected to higher forces during the impact cycle.

**Figure 17:**
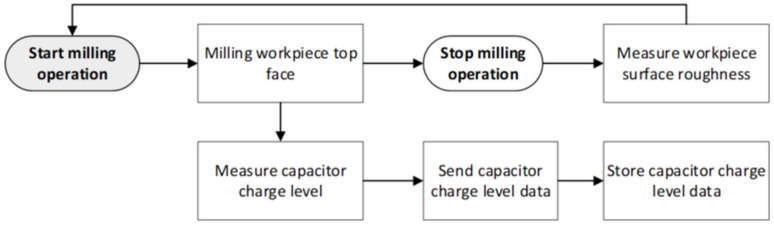
The flow chart of process steps used during experiment execution.

The next step of the experimental study investigated the ability of the proposed sensor node to detect gradual tool wear during milling operation. For this purpose, the device (Figure 13) was assembled with a sharp (new) four flute HSS end mill tool (see Table 4), which, according to the process parameters defined in Table 5, was used to machine the top surface of a 1.0037 type steel (see Table 3) workpiece, with a length=250 mm, height=50 mm and width=50 mm. The experimental study was carried out by machining the top surface of the workpiece 61 times continuously, starting with a sharp (new) end mill tool, gradually (over milling operation) achieving its wear. During the milling of the top surface of the workpiece, once the machining was started, data from the sensor node with the capacitor charge level were sent every 250 ms. A smartphone with Bluetooth connectivity was used for the receiver to visually display the data on the screen in real time and store it for later processing. Each time milling operation of the workpiece top face was completed, its surface roughness was measured and logged at 15 different points using Mitutoyo SJ-210 surface roughness tester (Mitutoyo America Corp., Aurora, IL, USA). A flowchart of the experimental process, showing the steps involved in each milling iteration carried out during the experiment, is given in Figure 17. Two parameters were recorded during the experiment: the capacitor charge level during continuous milling and the workpiece surface roughness measurements after each milling iteration. Both parameters recorded at the sensor node were fed as input data to an SVM-based prediction model to assess whether they can be used to detect the gradual tool wear in real time during milling operation, which is expressed by the relationship between the change of the capacitor charge level and the increase in workpiece surface roughness.

## 4. Experimental Results

### 4.1. Features’ Extraction for an SVM-Based Prediction Model

Support Vector Machines (SVMs) are one of the most popular supervised learning algorithms applied for both classification and regression problems [30]. The Support vector regression (SVR) approach is able to solve nonlinear problems with a comparably small number of model parameters. Unlike other machine learning algorithms, the algorithm does not suffer from the problem of overfitting [31]. Moreover, the SVR based prediction model is very suitable for edge devices due to its decision-making time. In the development of an intelligent monitoring system for the cutter wear process, the speed and robustness of the decision are the most important factors, because changes of the capacitor charge level can be observed within milliseconds. Since the effectiveness of an SVR depends upon the selection of kernels, the parameters of those kernels and soft margin parameter, different experiments have been carried out in this study.

Each milling iteration of the top surface of the workpiece lasted on average 10 min, during which 2400 data points were recorded to determine the charge level of the capacitor and 15 different surface points were taken to measure the average surface roughness after the milling operation. The average surface roughness values are considered as the output of the SVR model. However, the raw data representing the charge level of the capacitor, measured every 250 ms, are not suitable as input data for the model. Therefore, seven common statistical measures [32] have been calculated from the distribution of the capacitor charge level as provided in Table 6:

Feature *Avg*- is the simple average value of all 2400 data points, denoting capacitor charge level. Variation *Var* and standard deviation *Sd* are calculated accordingly.

The autocorrelation function (ACF) is a useful characteristic for finding recurring patterns. This characteristic indicates the degree of similarity between values of the same variables over two time intervals. This concept has been used for defining the attribute *ACorr*, which refers to the average autocorrelation value calculated between two measures of the capacitor charge level at times $x_{t}$ and $x_{t-k}$ [32]:

(3) $$ACorr=\frac{1}{n-1}\overset{n}{\underset{i\;=\;1}{\sum }}ACF(x_{i},x_{i-k}),\;k=1,2,3\ldots .$$

where value k—is the time interval (the lag), which represents autocorrelation between values that are one time interval apart.

The feature $M_{4}Avg$ calculates moving averages. In our case, four data points are taken and their average is calculated [33]:

(4) $$MAF\;=\;\frac{\left(x_{i}+x_{i+1}+\ldots +x_{i+\left(M-1\right)}\right)}{M}$$

(5) $$M_{4}Avg\;=\;\frac{1}{n-\left(M-1\right)}\overset{n-\left(M-1\right)}{\underset{i\;=\;0}{\sum }}MAF_{i}$$

where n—data points, where *M* is the size of the sliding window, and in our case M=4.

Another quite informative characteristic is interquartile *InterQ*, which calculates the difference between the third quartile and the first quartile for a data [33]:

(6) $$InterQ\;=\;Q_{3}-Q_{1}.$$

where $Q_{1}$—the first quartile, and $Q_{3}$—the third quartile.

Feature *Energy* is the sum of the squared data values [34]:

(7) $$Energy\;=\;\overset{n-1}{\underset{i\;=\;0}{\sum }}{\left(x_{i}\right)}^{2}$$

Three specific measures have been derived using expert’s knowledge:

- *Energy* provides the percentage amount of very high values of the capacitor charge level, $x_{i}>360,\;i=\bar{1,n}$. It has been noticed, that the amount of such values has a positive relationship with surface roughness and correlation coefficient is equal to 0.811.
- $Signal_{jump}$ provides the sum of squared differences ${\left(∆x_{i}\right)}^{2}$, including the condition: the value of ${\left(∆x_{i}\right)}^{2}\;$ has to be greater than 0.9 of the quantile of differences between data points, $Q_{∆x_{i}}\left(p\right),\;p\;=\;0.9$:
  (8) $$Signal_{jump}=\overset{n-1}{\underset{i=0}{\sum }}{\left(∆x_{i}\right)}^{2}>Q_{∆x_{i}}\left(0.9\right),{\;}^{}\;\mathrm{where}\;∆x_{i}=\left(x_{i+1}-x_{i}\right).\;$$
  where Q—quantile function, p—probability value 0<p<1. This feature highly correlates with the output (see Table 7).
- *Avg_cycle* is the average length of one capacitor charge cycle, until the set threshold level.

**Figure 18:**
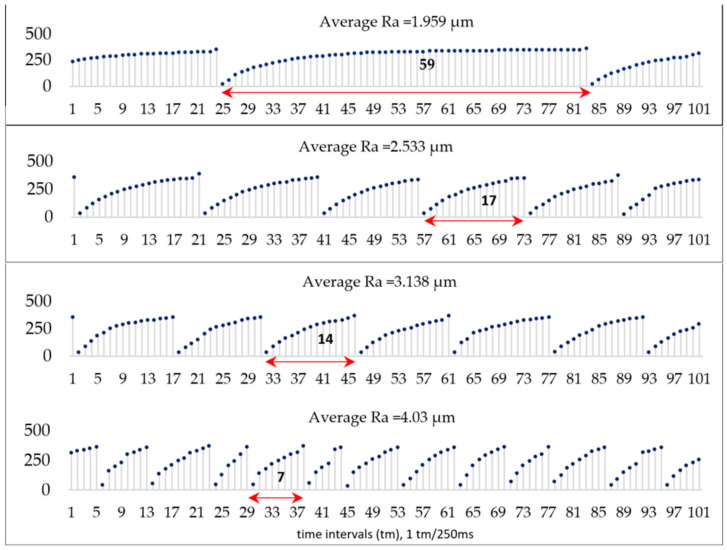
Capacitor charge cycle duration dependence on the surface roughness of the workpiece: 14.759 s vs. Ra = 1.959 µm, 4.25 s vs. Ra = 2.533 µm, 3.25 seconds vs. Ra = 3.138 µm, 1.75 seconds vs. Ra = 4.03 µm.

The end of the cycle is determined if the difference between data points is relatively large $∆x_{i}>h$. The most appropriate threshold value for h=150 has been determined experimentally. The average cycle length *Avg_cycle* is calculated by taking into account all recorded lengths at the capacitor charge level. It has been observed that higher values of workpiece surface roughness (Ra) have lower values of average capacitor charge cycle. For example, for a roughness of 1.959, the average capacitor cycle length is 59 time intervals (1 time interval = 250 ms), which is 59×250ms =14,750 ms =14.759 s, meanwhile for a roughness of more than 4, the cycle is very small averaging about 1.750 s. The relation between the decrease in the average capacitor charging cycle time and the increase in surface roughness is provided in Figure 18.

The obtained results show that there is a negative correlation (r=−0.743) between the length of the capacitor charging cycle and the surface roughness of the workpiece, which is due to the wear of the cutting edge of the milling tool. In this case, the charge level of the capacitor at the time of the measurement was expressed in integers, where one unit equals 0.0015 volts, and the MCU was set to discharge the capacitor when it reaches an integer value of 350, that is when its charge level equals 0.5 volts. During the milling operation, when the charge on the piezoelectric transducer capacitor voltage reaches or exceeds the set threshold value, the capacitor is discharged and the cycle repeats itself.

Ten features have been included for the prediction task and the correlation coefficients (see Table 7) show that the most informative features are *ACorr, InterQ, Energy, BigV* and $Signal_{jump}$. The most irrelevant feature (r=0.574) is the standard deviation of the capacitor charge level.

**Table 7:** Pearson correlation coefficient values.

	Avg	Var	Sd	ACorr	M_4_Avg	InterQ	Energy	BigV	Signal_jump_	Avg_cycle
Roughness	−0.739	0.641	0.574	−0.817	−0.767	0.825	0.812	0.811	0.889	−0.743

### 4.2. Model Evaluation Metrics and Prediction Accuracy Results

All modeling experiments were carried out using the Python programming language in Jupyter notebook in the Google Colab environment. The fit of the SVR model was evaluated by calculating the coefficient of determination and prediction error.

$R^{2}\;$(coefficient of determination) is commonly used to evaluate model performance [33].$\;R^{2}\;$ is the regression score, which is a statistical measure of how close the data are to the fitted regression line. In regression, it is a measure of how well the regression predictions approximate the real data. When $R^{2}$ equals to 1, it indicates that the regression predictions perfectly fit the data:

(9) $$R^{2}=\frac{SSR}{SST}=1-\frac{\sum_{i=1}^{m}{\left(y_{i}-\hat{y_{i}}\right)}^{2}}{\sum_{i=1}^{m}{\left(y_{i}-\bar{y}\right)}^{2}}$$

where *SSR* is the sum of squares of residuals, *SST*—the total sum of squares, $y_{i}$—the actual value, $\hat{y_{i}}$—the predicted value and $\bar{y}$ the mean value.

**Figure 19:**
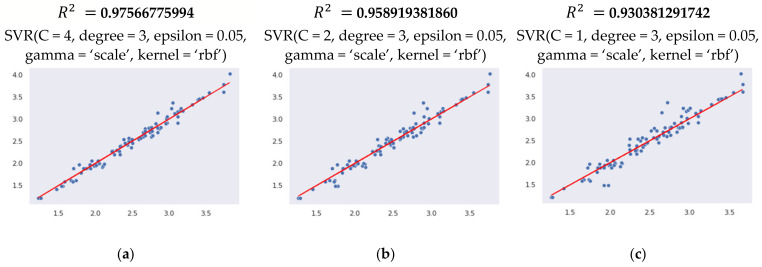

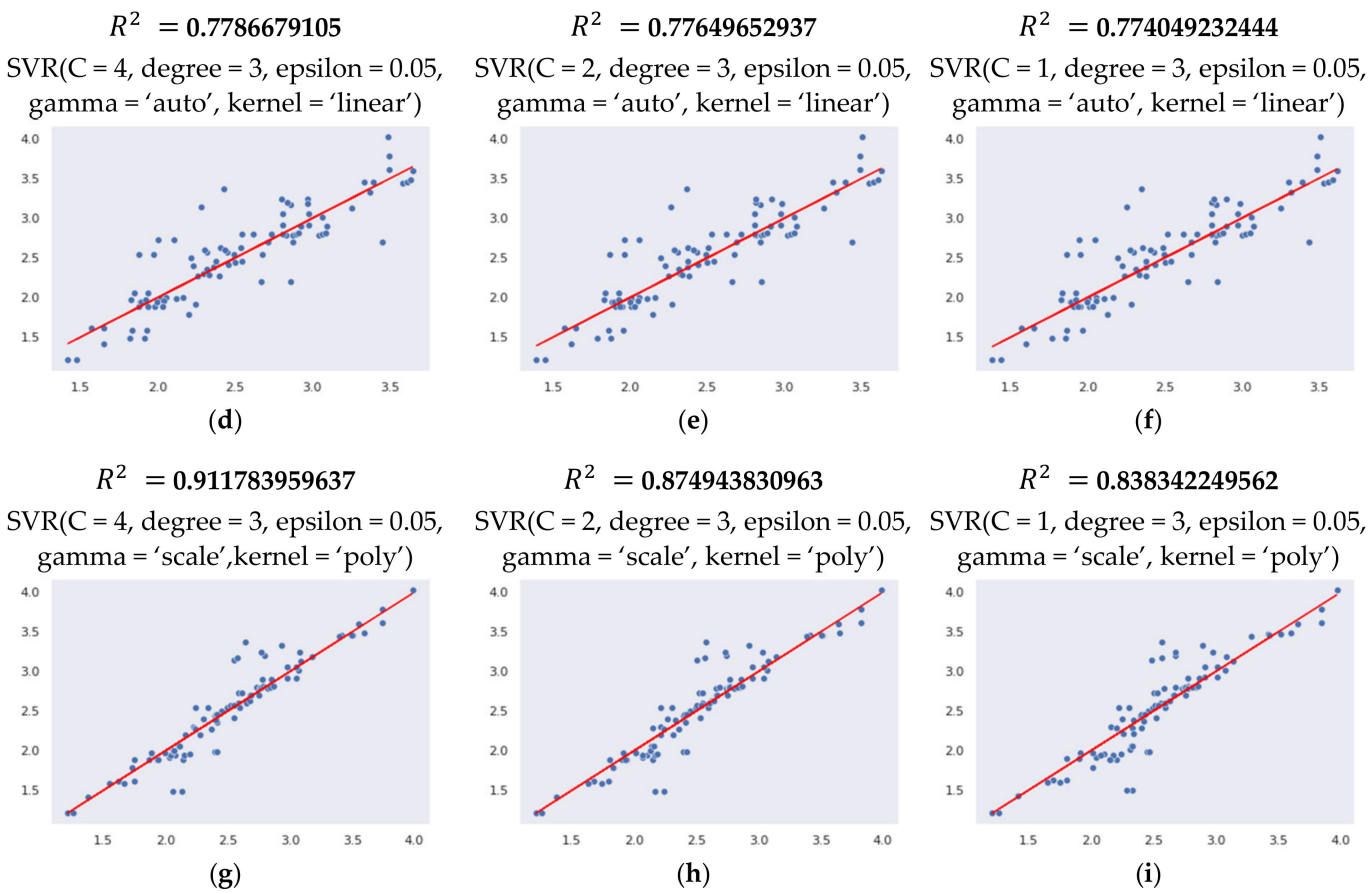
Coefficient of determination $\;R^{2}\;$ value for RBF-SVR model depending on the number and the type of kernels. (**a**)—4 rbf kernels, (**b**)—2 rbf kernels, (**c**)—1 rbf kernel, (**d**)—4 linear kernels, (**e**)—2 linear kernels, (**f**)—1 linear kernel, (**g**)—4 polynomial kernels, (**h**)—2 polynomial kernels, (**i**)—1 polynomial kernel.

The provided results (Figure 19) indicate that $R^{2}$ value for RBF-SVM model varies from 0.930 to 0.975, depending on the number of kernels, varying from 1 to 4. These results denote that the RBF-SVM model explains all the variability of the response data. More $R^{2}\;$ scoring variations can be observed with the polynomial SVM model, ranging from 0.838 to 0.911 respectively. The regression score of the linear SVM model is more or less stable at around 0.77.

Three error measures for time-series prediction are usually calculated: the root mean square error (RMSE); the mean absolute deviation (MAD) and the mean absolute percentage error (MAPE). In our experiments, MAPE is calculated to evaluate the prediction accuracy of SVM models. MAPE is a relative error measure that uses relative errors to compare the predicted accuracy between time-series models. The formula for calculating the MAPE is provided below [33]:

(10) $$E_{M}=\frac{1}{n}\overset{n}{\underset{i=1}{\sum }}\left|\frac{y_{i}-{\hat{y}}_{i}}{y_{i}}\right|\times 100$$

where *n*—the number of time point, $y_{i}$—is the actual value at a given time period *i*, and ${\hat{y}}_{i}$—is the predicted value.

**Figure 20:**
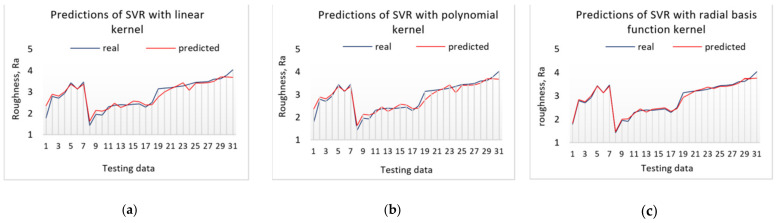
Testing results of the SVM model with different kernels: (**a**)—linear, (**b**)—polynomial, (**c**)—radial basis function.

The data used to test the model (capacitor charge level values over time) are obtained from 31 different milling operations. The average MAPE value of the SVM model with a radial basis function kernel and C = 4, predictions are equal to 2.420%. The SVM with a polynomial kernel and C = 4 resulted in an average MAPE value of 5.431%, while the highest error was observed with the linear kernel of 8.608%. The predicted and real (actual) values of the surface roughness during the testing are presented in Figure 20.

## 5. Discussion

As the data can be considered as a time series, various additional features such as entropy, “peak to peak” distance, seasonality and trend can be calculated for prediction.

The Seasonal-Trend Decomposition by Loess (STDL) method [34] can be applied to time series, because it can decompose a time series into seasonal, trend and remainder components [35]:

(11) $$Y_{t}=T_{t}+S_{t}+R_{t}.$$

where $T_{t}—$is the trend component, $S_{t}—$is the seasonal component representing for example the annual cycles, and $R_{t}$ is an irregular (remainder).

**Figure 21:**
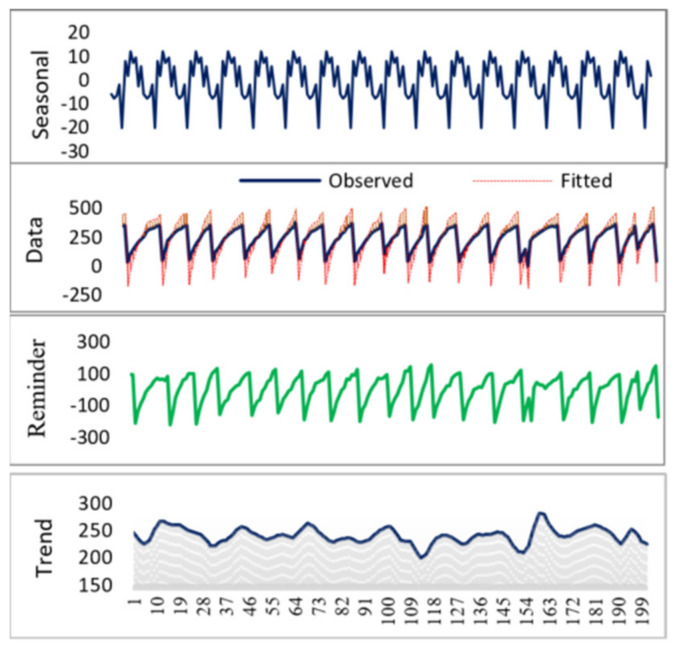
STDL of the capacitor charge level data, when workpiece surface roughness Ra = 4.03.

**Figure 22:**
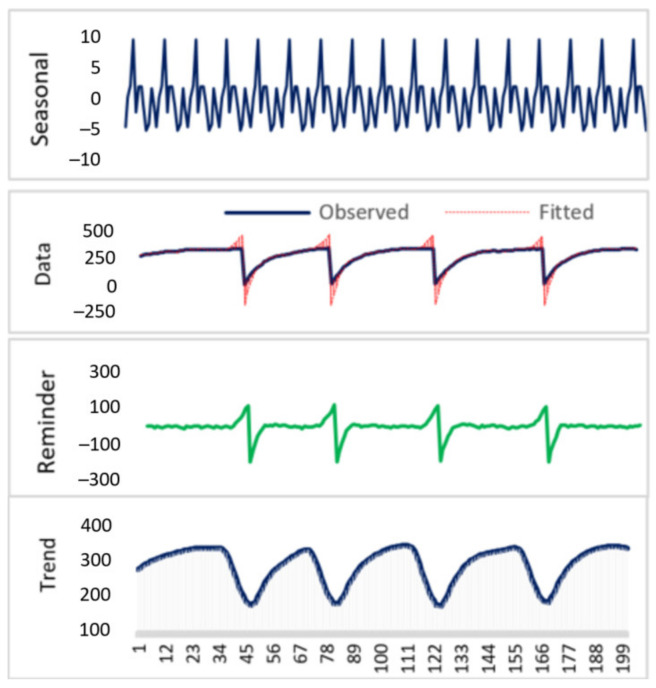
STDL of the capacitor charge level data, when workpiece surface roughness Ra = 3.21.

*STDL* model diagram for the capacitor charging level seasonal trend when workpiece surface roughness is Ra = 4.03 µm and Ra = 3.21 µm and dp = 200 (number of presented data points) is provided in Figure 21 and Figure 22, respectively. STDL parameters: seasonal period = 12, seasonal window = periodic, seasonal degree = 0, trend degree = 1, low pass degree = 1, robust loess fitting = False. The experimental results with different model parameters exhibit almost no seasonality, therefore we can conclude that the STDL model is not useful for our data analysis.

The feature *SE*—is the spectral Shannon entropy, often applied to time series [35]:

(12) $$SE=-\int_{-\pi }^{\pi }\hat{f}\left(\lambda \right)\;log\;\hat{f}\left(\lambda \right)d\lambda .\;$$

here $\hat{f}\left(\lambda \right)$ is an estimate of the spectral density of the data. It measures the predictability of the time series. Large *SE* values are calculated when the time series is difficult to forecast, while small values indicate a high signal-to-noise ratio.

Another popular time series feature is “peak-to-peak” which calculates the distance between two peaks: lowest and highest [32]:

(13) $$PtoP=\left|\max\left(X\right)-\min\left(X\right)\right|.\;$$

The entropy feature has provided promising results for our data, resulting in a significant value of correlation coefficient r=0.858. The peak-to-peak calculation is less informative and has an inverse correlation with the output value, r=−0.660.

**Figure 23:**
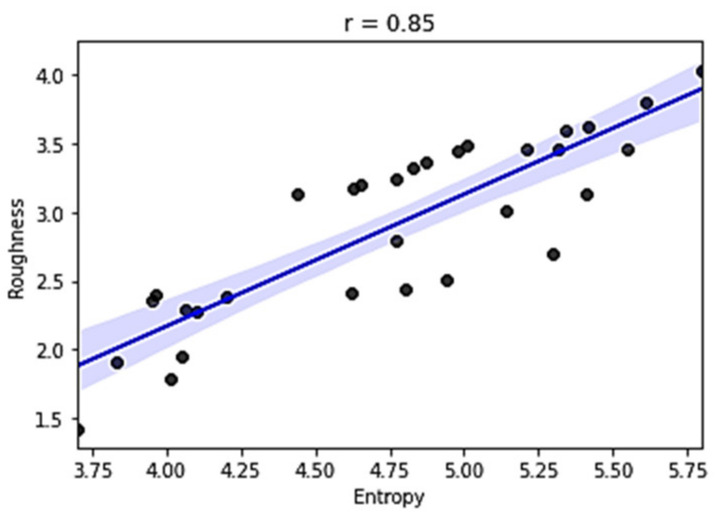
Relationship between data entropy value and roughness.

**Figure 24:**
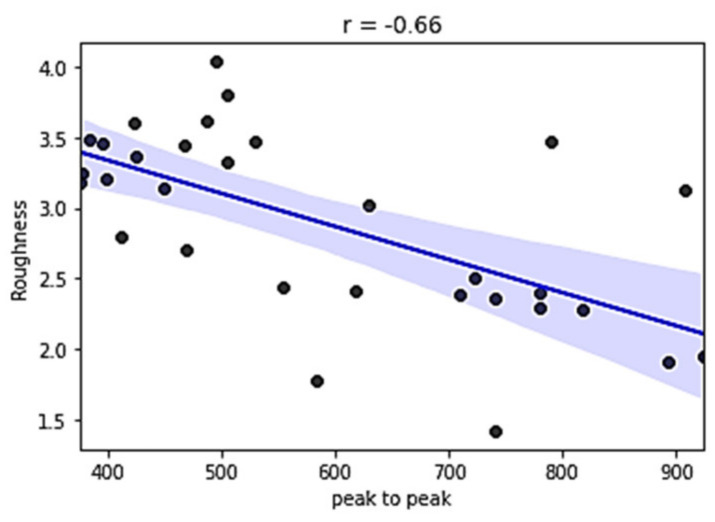
Relationship between data peak-to-peak value and roughness.

To visualize a linear relationship through regression, scatterplot diagrams of those two features (*SE* and *PtoP*) are provided in Figure 23 and Figure 24, including the regression line and the 95% confidence interval of that regression.

**Figure 25:**
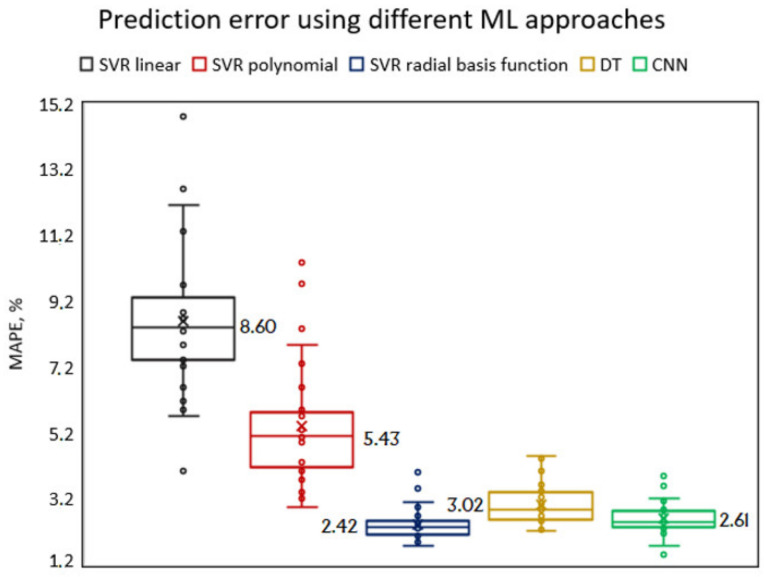
Comparison results for different ML algorithms MAPE (SVR with different kernels, DT and CNN) represented using boxplot.

Additional experimental investigations were performed by implementing other machine learning approaches. In particular, decision trees (regression) and convolutional neural networks were used to compare their performance with SVR on a selected dataset. A simple dense CNN architecture with a 5-layer dense block was selected [36], because the direct connection in the dense block can solve the problem of vanishing gradient, as it is less prone to overfitting compared to the deep CNN [31]. Furthermore, there is no need to use deep CNN architectures for image recognition, because our input features are numerical values (not tool wear images). The prediction results of the SVR different model, the decision tree and the CNN are provided below (Figure 25).

From the obtained results (Figure 25) we can conclude that SVM with radial basis function is the most accurate algorithm (MAPE error 2.42%), however the average MAPE error is only slightly different from the results of DT (3.02%) and CNN (2.61%), but the final decision should be made considering two factors: accuracy and performance speed. Convolutional neural networks have shown their superiority in terms of accuracy, however, the larger number of parameters and the complex architecture make this an extremely time-consuming approach. Besides, CNNs are more efficient in solving problems with a huge number of instances and attributes. For these reasons, the SVM model is preferable for this problem, noting that the prediction error is 7.28% lower than that of CNNs.

## 6. Conclusions

This study presents the design of a sensor node employing piezoelectric energy harvesting for wear detection in rotating shank-type tools. The results, obtained during modeling, revealed that the cone-shaped tool holder with helical slots introduces an L&T vibration mode coupling effect, which allows the torsional forces acting on the tool during cutting operation to be converted into longitudinal motion. The excited longitudinal motion can be used to deform the piezoelectric transducer generating the voltage.

- The performed FEM studies of a tool holder with a piezoelectric transducer show that a tool holder with helical slots, experiences more than two times higher surface displacement amplitudes in longitudinal direction, when it is excited in the L&T mode.
- A tool holder with helical slots, when assembled with piezoelectric transducer (and excited to resonate at L&T mode), produces more than two times the voltage compared to a tool holder without helical slots.
- The experimental studies have confirmed the FEM modeling results, where the excited tool holder with helical slots has more than 2 times higher surface displacement amplitudes in the longitudinal direction and is able to generate more than 3 times higher amount of voltage from the embedded piezoelectric transducer during milling operation compared to a tool holder without helical slots.
- The sufficient power generated by the device allows it to be used as a wireless sensor node, that can be used in milling operations for detecting the wear of the end mill tool, when the voltage generated by the piezoelectric transducer increases exponentially due to the progressive cutter degradation.
- The machine learning approach was applied to solve the milling wear prediction problem using surface roughness measurements as the key indicator of tool condition. SVR with a radial basis function kernel provides the lowest prediction error (2.420% MAPE) compared to polynomial (5.431% MAPE) and linear (8.608% MAPE) kernels. However compared to other ML methods, namely CNN and DT, the superiority of SVR-RBF is not so apparent.
- By exploring the computed empirical features of the SVR model, it was observed that time series features such as autocorrelation, interquartile, absolute energy, entropy are the most relevant for solving the problem. However, according to the correlation coefficient, the most informative feature is the specially created feature $Signal_{jump}$ (r=0.889) used for determining signals’ jumps due to the difference in data points at the 90% confidence level.

## 7. Patents

After obtaining positive results during the experimental research, a patent application entitled: “Wireless sensor to assess the quality of rotating tools” has been submitted for the developed device to The State Patent Bureau of the Republic of Lithuania.

## Figures and Tables

**Table 1:** Chemical composition and mechanical properties of C45 (EN 1.0503) type steel.

**Chemical Composition, %**							
C	Si	Mn	Ni	P	S	Cr	Mo
0.43–0.5	Max. 0.4	0.5–0.8	max. 0.4	max. 0.045	max. 0.045	max. 0.4	max. 0.1
**Physical Properties**							
Brinell hardness		Young’s modulus		Poissons ratio		Density	
180		210 Gpa			0.3	7800 kg/m^3^	

**Table 2:** Components constituting a condition monitoring device for a rotating shank type tool.

Number	Component Description
1	Holder’s Morse cone for assembly inside CNC center
2	Antenna for wireless data transmission
3	PCBA holder inside tool holder
4	PCBA with data processing and transmission components
5	Back-mass
6	Stack type piezoelectric transducer
7	Flange for assembling tool holder with Morse cone cover
8	Cone shaped tool holder with helical slots
9	End mill tool

**Table 3:** Chemical composition and mechanical properties of 1.0037 type steel.

**Chemical Composition, %**				
C	Mn	P		S
0.17–0.20	1.40	0.045		0.045
**Physical Properties**				
Brinell hardness	Young’s modulus	Poissons ratio		Density
324	200 GPa		0.29	7700 kg/m^3^

**Table 4:** The main parameters of the end mill tool used during the machining operation.

Tool type	HSS
End type	Straight
Number of teeth, Z	Four
Helix angle	35°
Shank diameter	10 mm
Cutting part diameter	10 mm
Shank diameter	10 mm
Working part length	25 mm
Overall length	75 mm

**Table 5:** Milling process parameters used during experiment.

Parameter	Spindle Speed, n	Feed Speed, v_f_	Feed Per Tooth, f_z_	Axial Depth of Cut, a_p_	Radial Depth of Cut, a_e_
Value	1210 RPM	148 mm/min	0.031 mm/tooth	1 mm	9.8 mm

**Table 6:** Calculated statistical features used as SVR model input data.

Feature Name	Explanation
*Avg*	Average value of the capacitor charge level values
*Var*	Variability value of the capacitor charge level values
*Sd*	Standard deviation of the capacitor charge level values
*ACorr*	Autocerrelation value of the capacitor charge level values
$M_{4}Avg$	4 data point simple moving averages of the capacitor charge level values
*InterQ*	Interquartile value of the capacitor charge level values
*Energy*	Absolute energy of the capacitor charge level values

## Nomenclature

*SVM*	Support vector machine
*TCM*	Tool condition monitoring
*MCU*	Microcontroller
*ML*	Machine learning
*RF*	Random forest
*RBF*	Radial Basis Function
*MAPE*	Mean absolute percentage error
*L&T*	Longitudinal and torsional composite vibrational mode
*CAD*	Computer aided design
*L_f_*	Free length of the end mill (mm)
*FEM*	Finite element method
*PZT*	Piezoelectric transducer
*OD*	Outer diameter (mm)
*ID*	Inner diameter (mm)
*H*	Height (mm)
*PCBA*	Printed circuit board assembly
*MCU*	Microcontroller
*DC*	Direct current
*3D*	Three dimensional
*CNC*	Computerized numerical control
*HSS*	High speed steel
*n*	Spindle speed (RPM)
*v_f_*	Feed seed (mm/min)
*f_z_*	Feed per tooth (mm/tooth)
*a_p_*	Axial depth of cut (mm)
*a_e_*	Radial depth of cut
u_K_, u_N_	node displacement vectors
*M*	Mass matrix
*K*	Stiffness matrix
*C*	Damping matrix
α	Mass proportional damping coefficient
β	Stiffness proportional damping coefficient
*C*	Carbon (%)
*Si*	Silicon (%)
*Mn*	Manganese (%)
*Ni*	Nickel (%)
*P*	Phosphorus (%)
*S*	Sulfur (%)
*Cr*	Chromium (%)
*Mo*	Molybdenum (%)
*Ra*	Arithmetic mean roughness (µm)
*Avg*	Average value of the capacitor charge level values
*Var*	Variability value of the capacitor charge level values
*Sd*	Standard deviation of the capacitor charge level values
*ACorr*	Autocorrelation value of the capacitor charge level values
*M*_4_ *Avg*	4 data point simple moving averages of the capacitor charge level
*InterQ*	Interquartile value of the capacitor charge level values
*Energy*	Absolute energy of the capacitor charge level values
*ACF*	Autocorrelation function
*MAF*	Moving average formula
*k*	Time interval
*Q*	Quantile function
*Q*_1_	First quartile
*Q*_4_	Fourth quartile
*p*	Probability value (0 < *p* < 1)
*r*	Negative correlation
$R^{2}$	Coefficient of determination (0 < $R^{2}$ ≤ 1)
*SSR*	Sum of squares of residuals
*SST*	Total sum of squares
$\hat{y_{i}}$	The predicted value
$\bar{y}$	The mean value
PtoP	Peak-to-peak
MAPE	The root mean square error
*STDL*	Seasonal-Trend decomposition by losses
$T_{t}$	Trend component
$S_{t}$	Seasonal component
$R_{t}$	Irregular
*SE*	Shannon entropy
$\hat{f}\left(\lambda \right)$	Estimate of the spectral density of data
